# Elucidating the Role of Chromatin State and Transcription Factors on the Regulation of the Yeast Metabolic Cycle: A Multi-Omic Integrative Approach

**DOI:** 10.3389/fgene.2018.00578

**Published:** 2018-11-30

**Authors:** Víctor Sánchez-Gaya, Salvador Casaní-Galdón, Manuel Ugidos, Zheng Kuang, Jane Mellor, Ana Conesa, Sonia Tarazona

**Affiliations:** ^1^Genomics of Gene Expression Laboratory Centro de Investigación Príncipe Felipe, Valencia, Spain; ^2^BioBam Bioinformatics S.L., Valencia, Spain; ^3^Institute for Systems Genetics, NYU Langone Health, New York, NY, United States; ^4^Department of Biochemistry University of Oxford, Oxford, United Kingdom; ^5^Microbiology and Cell Science Department, Institute for Food and Agricultural Research, University of Florida, Gainesville, FL, United States; ^6^Genetics Institute, University of Florida, Gainesville, FL, United States; ^7^Applied Statistics, Operational Research and Quality Department Polytechnic University of Valencia, Valencia, Spain

**Keywords:** YMC, histone modifications, gene expression, omics integration, regression

## Abstract

The Yeast Metabolic Cycle (YMC) is a model system in which levels of around 60% of the yeast transcripts cycle over time. The spatial and temporal resolution provided by the YMC has revealed that changes in the yeast metabolic landscape and chromatin status can be related to cycling gene expression. However, the interplay between histone modifications and transcription factor activity during the YMC is still poorly understood. Here we apply an innovative statistical approach to integrate chromatin state (ChIP-seq) and gene expression (RNA-seq) data to investigate the transcriptional control during the YMC. By using the multivariate regression models N-PLS (Partial Least Squares) and MORE (Multi-Omics REgulation) methodologies, we assessed the contribution of histone marks and transcription factors to the regulation of gene expression in the YMC. We found that H3K18ac and H3K9ac were the most important histone modifications, whereas Sfp1, Hfi1, Pip2, Mig2, and Yhp1 emerged as the most relevant transcription factors. A significant association in the co-regulation of gene expression was found between H3K18ac and the transcription factors Pip2 (involved in fatty acids metabolism), Xbp1 (cyclin implicated in the regulation of carbohydrate and amino acid metabolism), and Hfi1 (involved in the formation of the SAGA complex). These results evidence the crucial role of histone lysine acetylation levels in the regulation of gene expression in the YMC through the coordinated action of transcription factors and lysine acetyltransferases.

## 1. Introduction

The Yeast Metabolic Cycle (YMC) is defined by robust periodic oscillations of gene expression that appear in continuous culture systems under aerobic glucose-limited conditions. These cycles last about 4–5 h and exhibit respiratory oscillations alternating between periods of high oxygen consumption (HOC) and low oxygen consumption (LOC), and factors as glucose concentration and external stimuli can affect period length and amplitude (Klevecz et al., 2004; Tu et al., 2005). The nature of the YMC has been extensively studied and is associated with other biological rhythms such as redox cycles and the cell cycle (Mellor, 2016). However, there are still many aspects of the YMC that are unknown or poorly understood due to the complexity of the molecular connections that coordinate the cellular metabolic state, molecular landscape and physiological response during cycling.

Gene expression during the YMC has been characterized using microarrays (Klevecz et al., 2004; Tu et al., 2005; Slavov et al., 2011) and RNA-Seq (Kuang et al., 2014). Transcriptional analyses identified three main phases of expression during the YMC: an Oxidative phase (OX), a Building phase (RB) and a Charging phase (RC). The RB phase was first defined as a reductive phase (Tu et al., 2005), but recent works (Murray et al., 2007; Mellor, 2016) have highlighted its oxidative state and proposed it to be part of the HOC phase. Functional profiling of these phases revealed an orchestration of gene expression, which fluctuates in response to environmental conditions, drives the cellular physiological changes and prepares the molecular mechanisms necessary for cycling. Metabolomics studies have shown that metabolite profiles also follow a periodic cycle across the YMC (Tu et al., 2007; Mohler et al., 2008), highlighting their importance in enzymatic allosteric regulation and synchronization of yeast ultradian rhythms (Mellor, 2016).

Cycling of histone modifications during the YMC confirms that they constitute cellular sensors of the metabolic conditions. For example, cycling levels of acetyl-CoA (a cofactor for histone acetylation) reflect alternative high and low energy states of yeast cells (Cai et al., 2011) and might be key to coordinate gene expression (Kuang et al., 2014). Kuang and co-authors showed that chromatin changes have a temporal association with transcripts, as both present similar oscillations. They correlated gene expression clusters with histone modifications to reveal the contribution of each histone modification to the regulation of the different YMC phases, and showed sequential regulation of genes involved in transcription, mitochondrial activity, cell cycle and different metabolic processes along the YMC. However, in their study, no significant relationships were established between histone modifications and the expression of specific genes or transcriptional regulators, which are still open questions regarding the functional orchestration of the system.

Few studies have investigated the potential role of transcription factors (TFs) in the regulation of the YMC. Rao and Pellegrini (2011) analyzed the periodic activities of TFs to explain the regulation of the YMC phases, while Kuang et al. (2017) inferred the spatio-temporal DNA binding of important TFs across the cycle. While it is reasonable to assume that regulation during the YMC consists of a combination of histone modifications coupled with TF control, these two regulatory layers have never been jointly studied. Such combined analysis would help to understand the contribution of each regulatory layer to the transcriptional dynamics observed in the YMC, and to decipher the significance of the interaction between specific histone marks and TFs in controlling gene expression.

In order to shed light into the regulatory mechanisms behind the YMC, we present here a novel strategy for the integrative analysis of the chromatin state and gene expression in this process. We used data from Kuang et al. (2014), that contains ChIP-seq experiments for 8 different histone modifications and matching RNA-seq data. In addition, we included a ChIP-seq dataset on an additional histone modification (H3K18ac), which turned out to be a key regulator of YMC. We specifically analyzed the interplay between chromatin status, transcription factor binding and gene expression, and identified a core set of TFs that are relevant to the synchronization between histone marks and transcriptional regulation. Our results revealed the impact of the different histone modifications on YMC progress and pointed out different TFs that might contribute to the molecular regulation of the cycle. Overall, this integrative analysis unravels regulatory mechanisms controlling switches in cellular processes that allow yeast to respond to factors affecting the metabolic cycle.

## 2. Materials and Methods

### 2.1. Omic Data Sets

Gene expression and histone modification data from Kuang et al. (2014) were retrieved from the GEO repository (accession number GSE52339). We also included in our study an additional histone modification ChIP-seq experiment for H3K18ac, provided by Dr. Mellor's laboratory, which was uploaded to GEO repository with accession number GSE118889. We complemented Kuang's data with this histone modification because of its relevance in transcriptional regulation (Cui et al., 2011; Weiner et al., 2015). All omic measurements were obtained from YMC experiments as described in Tu et al. (2005). As the duration of the different phases of the YMC is not the same, samples were unevenly taken at each phase to result in an equal number of time points in each phase of the cycle (Kuang et al., 2014). The number of sampling points was 16 for both RNA-seq and histone modification data.

Gene expression was measured by RNA-seq, Illumina HiSeq 2000, single-end reads, 50 bp long, at 16 sampling points. RNA-seq reads were pre-processed as in Kuang et al. (2014), where expression data had been normalized by sequencing depth, log-transformed and centered per gene. Histone modifications were measured by ChIP-seq using antibodies against eight different marks: H4K16ac, H3K36me3, H3K4me3, H4K5ac, H3K9ac, H3K56ac, H3K14ac, H3K18ac, and H3 that was used as a control. These data had also been collected at 16 time-points along the YMC. 10 biological samples per time point were obtained in two different batches, and H3K9ac was measured in both of them. H3K18ac samples were extracted in one of these batches. The samples were sequenced using three different ChIP-seq technologies (Illumina HiSeq 2000 ChIP, Illumina Genome Analyzer ChIP, and AB SOLiD System), which provided different read lengths from 35 to 51 bps. ChIP-seq data were analyzed as detailed in next sections.

### 2.2. ChIP-Seq Data Pre-Processing

The quality of ChIP-seq fastq files was assessed with FastQC software (http://www.bioinformatics.babraham.ac.uk/projects/fastqc/). Reads were quality-filtered and trimmed to discard low quality reads before mapping to the reference genome. The Trimmomatic software v0.32 (Bolger et al., 2014) was applied with more restrictive filters for the highest quality sequencing samples. Minimum quality was set to 30 (restrictive) or 25 (non-restrictive) at the beginning and end of the read, with a sliding window of 5 and a minimum length of 28 bp (restrictive) or 25 bp (non-restrictive). As a result, H3K36me3 and H4K16ac histone modifications were discarded due to their low sequencing quality.

Sequencing reads for the remaining histone modifications were mapped to the Ensembl database (release 91) *S. cerevisiae* reference genome (Langmead et al., 2009) using Bowtie (Zerbino et al., 2017). Multimapped reads were discarded. For AB SOLiD reads, reference genome was first converted to color space coding using bowtie-build with parameter –*c*. Duplicated reads were removed with Samtools software (Li et al., 2009).

### 2.3. ChIP-Seq Quantification

In order to obtain ChIP-seq quantification values, coverage per nucleotide was calculated for the whole genome with the program genomecov from the bedtools suite (Quinlan and Hall, 2010), specifying the parameter –*d*. After an exploratory analysis of the coverage across different genomic regions, we defined two genomic regions per histone modification and gene, corresponding to either the 300 bp upstream or downstream regions from the gene transcription start site (TSS). For each gene and region, the average of the coverage was computed using in-house Python scripts and the yeast genome annotation (gtf file) obtained from Ensembl (release 91) (Zerbino et al., 2017). Consequently, two quantification matrices were generated for each histone modification, one for upstream and one for downstream data.

The H3 ChIP-seq data were used as control to normalize quantification values. Each histone modification sample was first corrected by its sequencing depth, then divided by the H3 sample at matching time point, log-transformed and centered. This normalization was proven to be effective to remove batch effects as verified by histone modification H3K9ac (measured in both batches). After this correction, the H3K9ac dataset with the highest quality was selected for further analyses.

### 2.4. Differential Expression and Clustering

Differential expression analysis of RNA-seq data was performed with R maSigPro package (Conesa et al., 2006; Nueda et al., 2014), which applies a polynomial regression model to analyse time-course gene expression data. A polynomial degree of 3 was selected as this provided the model with the highest adjusted *R*^2^ (results not shown). Differentially expressed genes (DEGs) were called by having a significant model (False Discovery Rate (FDR) adjusted *p*-value < 0.05) and a minimum *R*^2^ value of 0.6. DEGs were clustered within maSigPro using the k-means method and setting the total number of clusters to 3.

### 2.5. N-PLS

Partial Least Squares regression (PLS) is a multivariate regression method commonly used to study the relationship between a response matrix and a predictor matrix (Wold et al., 1983; Geladi and Kowalski, 1986). These two matrices have two dimensions which are, typically, observations (e.g., time points) in rows and variables (e.g., genes) in columns. However, in some studies, a third dimension could be considered when having several treatments, different experiments, etc. In our case, the different histone modifications can constitute the third dimension, for instance. For these 3-dimensional structures, a multi-way extension of PLS method called N-PLS (Bro, 1996) can be applied. In N-PLS, the dimensions are called modes so, in our data, we will have three modes: first for genes, second for time-points and third for histone modifications.

Both PLS and N-PLS approaches are considered dimension reduction techniques because they identify a set of new variables (components) that are a linear combination of the original variables maximizing the covariance between the response and predictor matrices. Therefore, high-dimensional data can be graphically displayed in a much lower dimension space. The loadings are defined as the projections of the old variables, observations, etc. on each of the components of the new low dimensional space. The interpretation of these components is achieved through the information provided by the so-called core matrix, which indicates which combinations of components from the different modes explain most of the variability of the response dataset.

Up to our knowledge, there is no R library available to compute N-PLS models on data where the response Y is a bidimensional matrix (genes and time points in our case), while the predictor X structure is 3-dimensional (genes, time points and histone modifications). Thus, we programmed ourselves the N-PLS method following the algorithm described in Bro (1996).

### 2.6. MORE Regression Models

MORE (Multi-Omics REgulation) (https://bitbucket.org/ConesaLab/more) is an R method for omics integration based on Generalized Linear Models (GLMs) that aims to explain gene expression as a function of potential gene regulatory elements. MORE can identify the most relevant gene regulators from a large subset of putative regulators, by finding those that significantly associate to changes in their expression levels. MORE first filters out the regulators with low variability and reduces multicollinearity by grouping highly correlated regulators, Secondly, two different variable selection procedures are applied: Elastic Net penalized regression (Zou and Hastie, 2005) and stepwise regression (Draper and Smith, 2014), and a final regression model is then provided for each gene.

We generated two MORE regression models for each DEG. The first model included histone modifications as potential regulators. In this case, the predictors for each gene were the two regions defined for each of the six considered histone modifications, resulting in an initial model that included 12 regulatory variables. The second MORE model considered differentially expressed TFs as predictors. The potential target genes of TFs were retrieved from the Yeastract database (Teixeira et al., 2017). After model adjustment, significant regulators of genes were selected by having regression coefficient p-values < 0.05. The final result of this analysis is a gene-histone mark/TF association file indicating if there is a significant relationship between the gene and the regulatory factor.

### 2.7. Functional Enrichment Analysis

In order to evaluate the functional impact of each histone mark or TF in relation with the different phases of the YMC, we performed a functional enrichment analysis on the set of genes significantly regulated by each regulatory factor as obtained from MORE models. Functional terms were retrieved from Gene Ontology (GO) (Ashburner et al., 2000; Consortium, 2016) and from Kyoto Encyclopedia of Genes and Genomes (KEGG) (Kanehisa and Goto, 2000; Kanehisa et al., 2016) databases, and a Fisher's Exact test was applied to select the significantly enriched functional categories (*p*-value < 0.05). The genes regulated by each histone modification were grouped according to the YMC cluster they belong to and the functional enrichment analysis was performed on each of the three groups. For TFs, the number of regulated genes was much lower and the enrichment analysis was done without YMC phases separation.

### 2.8. Selection of the Most Relevant Regulations

The MORE analysis returns a large number of significant factors involved in gene regulation. An enrichment analysis approach was applied to select most relevant regulators during the YMC. We considered a TF to be relevant if the proportion of MORE significant models involving this TF was significantly higher than the proportion of the TF regulations in Yeastract database. Additionally, we selected most relevant histone modification-TF pairs as those co-regulating a proportion of genes higher than expected by chance. Both analyses were done with a Fisher's Exact test (FDR adjusted *p*-value < 0.05, and odds ratio > 1).

## 3. Results

### 3.1. Time Point Alignment

**Figure 1:**
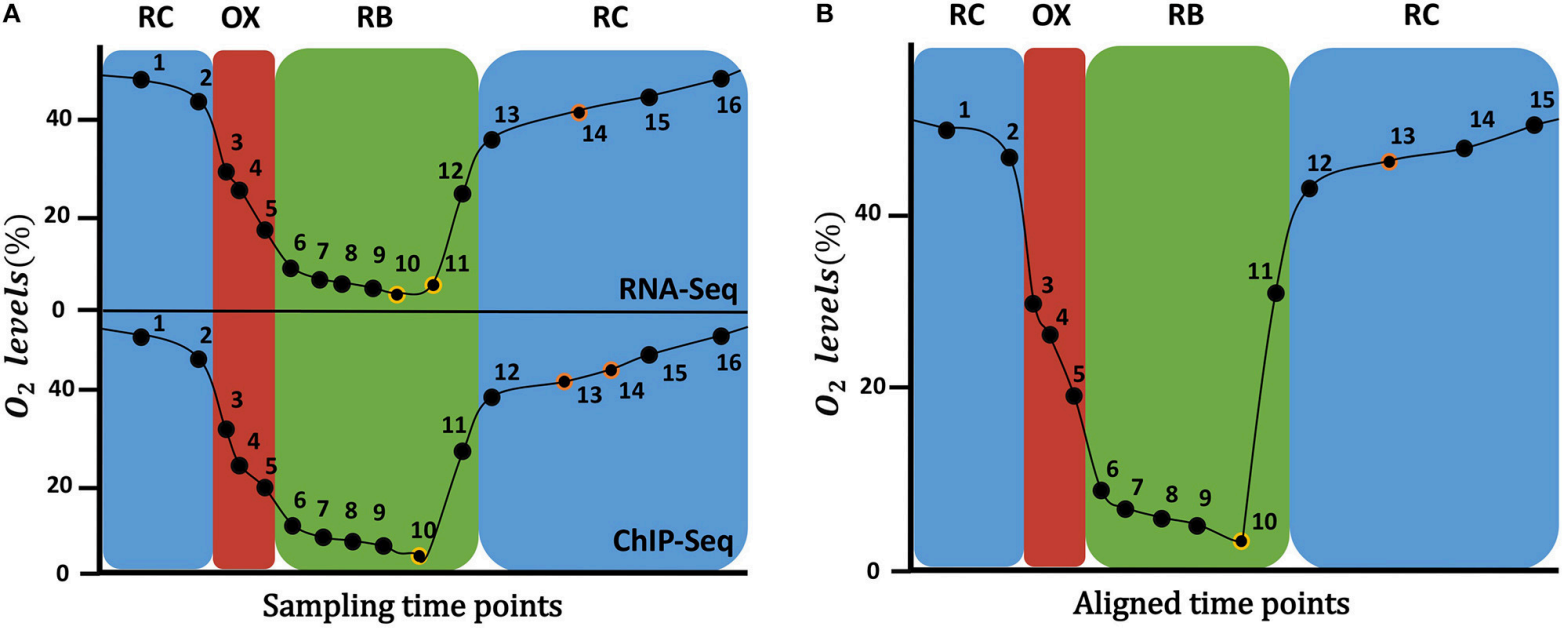
Time points sampled during the YMC. On the **(A)**, the original time points sampled for RNA-Seq data (top) and ChIP-Seq data (bottom) at each YMC phase are shown. On the **(B)**, the 15 time points after the alignment of the two time series are displayed: time-points 10 and 11 from RNA-seq were averaged, as well as time-points 13 and 14 from ChIP-seq. On the Y axis the percentage of oxygen in the environment is indicated.

Kuang et al. (2014) measured gene expression and histone modifications at 16 sampling points as shown in Figure 1A, where it can be observed that not all the time points are equivalent for both data types. For instance, RNA-seq RB phase has 7 time-points, while ChIP-seq has only 6 time-points. Since the omics integrative analysis requires matchable observations, that is, the same time points for both types of data, we had to “align” them. For that, oxygen consumption levels were used as a cellular metabolic state indicator to compare the two experiments and match disagreeing time-points. As a result, time-points 10 and 11 from RNA-seq were averaged, as well as time-points 13 and 14 from ChIP-seq, resulting in final data matrices with 15 rather than the original 16 time points (Figure 1B).

### 3.2. Gene Clustering Into YMC Phases

First of all, we selected the genes with significant changes across YMC and we clustered them according to their temporal profile in order to assign them to one of the YMC phases. For that, we applied the maSigPro R package, which returned the differentially expressed genes (DEGs) and clustered them. A total of 2,552 out of the 5,992 genes in the initial RNA-seq dataset were declared as DEGs. Principal Component Analysis (PCA) on these DEGs showed a clear separation of the time points according to the different metabolic phases of the YMC (Supplementary Figure 1), which corroborated the good quality of the data and of the differential expression results.

The 2,552 DEGs were clustered according to their temporal profiles into three groups to recapitulate the currently defined phases of the YMC (Tu et al., 2005; Kuang et al., 2014). The number of genes assigned to each of the clusters was 1428, 426, and 698, respectively (Figure 3). Clusters were assigned to their corresponding phase by matching expression peaks from the gene profiles to their position in the time-course. We assessed the quality of our clustering results with the Silhouette metric, that indicates how well each gene fits in the assigned cluster, and compared to the previously reported clusters in Kuang et al. (2014). While clusters in our study and in Kuang et al. (2014) largely agreed, a better clustering performance was obtained with our analysis strategy (Supplementary Figure 2).

### 3.3. Expression Changes in YMC Were Mostly Driven by H3K9ac And H3K18ac

**Figure 2:**
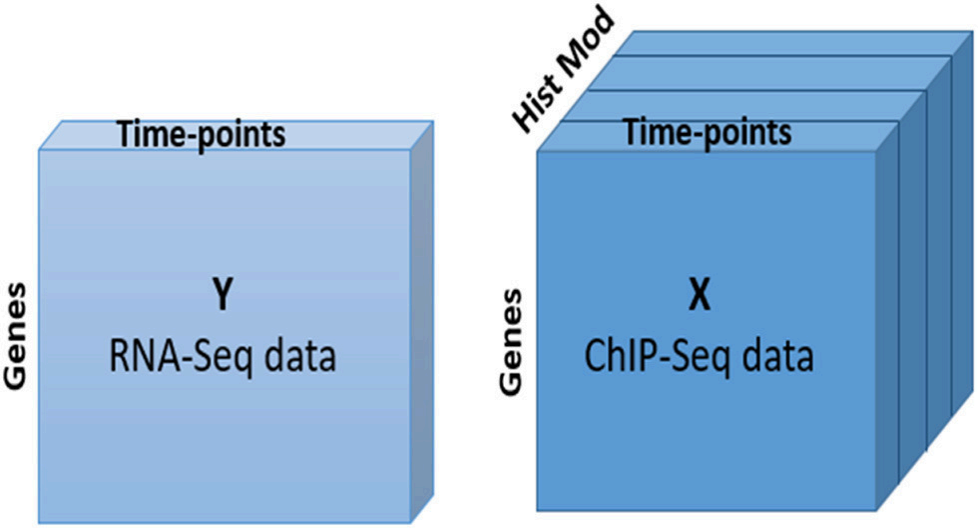
N-PLS model. Structure of response (Y) and predictor (X) matrices.

After retrieving the histone modification data for DEGs, we applied the N-PLS regression model, which allowed us to integratively explore the relationship between gene expression and chromatin status from a global perspective and to assess the quality of the multi-omic data set. In our analysis, RNA-seq data, with two dimensions or modes (genes and time points), was used as response matrix. ChIP-seq data was taken as the predictor variable. This dataset consists of three dimensions or modes: two of them are coincident with RNA-seq and the third mode captures the different histone modifications and genomic regions (Figure 2).

**Figure 3:**
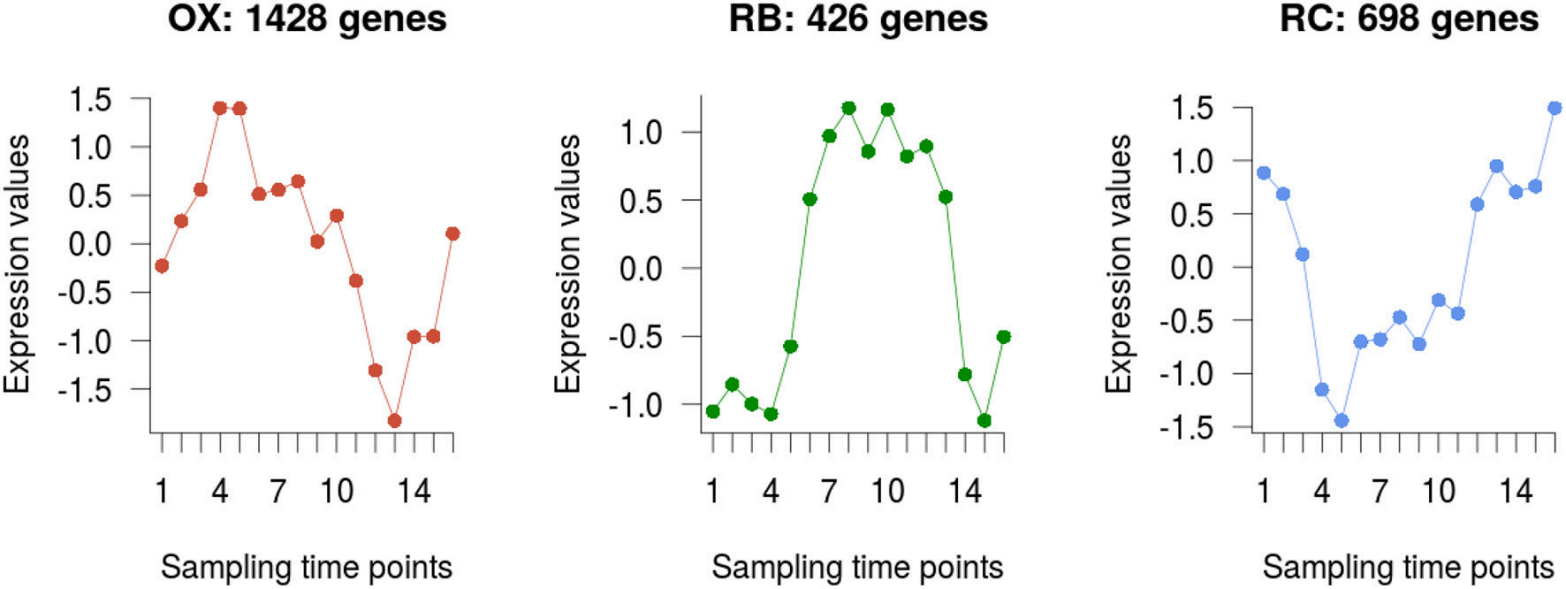
Genes clustering. Average profile of the differentially expressed genes in each of the three clusters obtained, which are in turn associated to each YMC phase.

**Figure 4:**
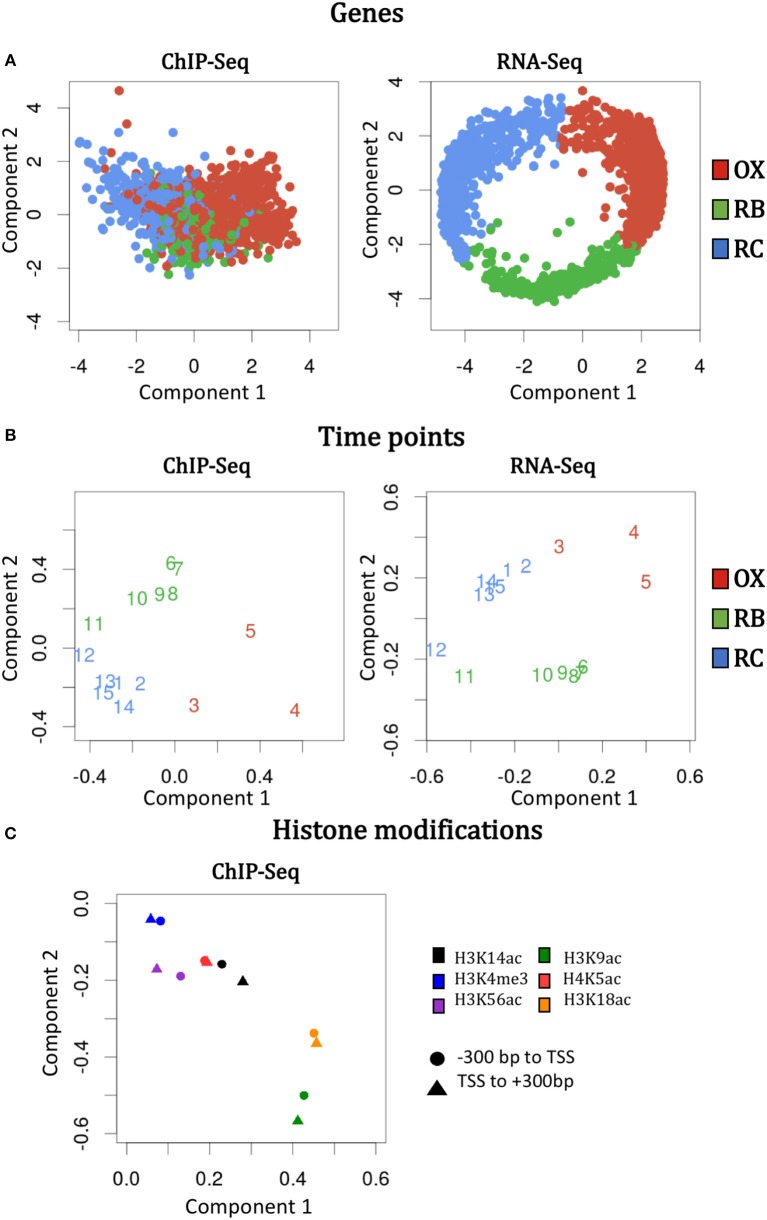
N-PLS loading plots for each mode and omic data type. The loading values are the original values of genes, time points and histone modifications projected onto the new spaced formed by component 1 (X axis) and 2 (Y axis). Left and right columns display the information for ChIP-seq and RNA-Seq data, respectively. **(A)** Genes loadings. **(B)** Time points loadings. **(C)** Histone modifications loadings.

The idea of N-PLS method is to generate new variables (components) that are linear combinations of the original variables and collect most of the covariance between the response and the predictors. In this case, we selected a model with two components that are represented in the X and Y axes of Figure 2, respectively. To understand the relevance of these components and their relationship with the original modes (genes, time points and histone modifications), we must look at the core matrix (Supplementary Table 1). The first element of the N-PLS core revealed that the combination of the second components for each of the three modes (genes, time points and histone modifications) captured most (60.7%) of the gene expression variability. The second most important core element connected component 2 of the first and second modes (genes and time points), with component 1 for the third mode (histone modifications), explaining 23.9% of the variability in the data. Therefore, more than 80% of the variance was explained with these two elements of the core. Figures 4A–C show the loading plots for the three modes, that is, the projections of genes, time points and histone modifications onto the new space formed by the N-PLS components.

N-PLS loading plots of mode 1 (genes) and mode 2 (time points) at both RNA-seq and ChIP-seq followed the phases of the YMC, as expected (Figures 4A,B). The second component of the second mode separated time-points 6 to 11 from time-points 1 to 5 and 13 to 15, which correspond to high and low oxygen consumption stages of the YMC, respectively. The second component of the first mode showed separation of genes at RB phase from genes associated to OX and RC phases, and that these differences were more pronounced in RNA-seq than in ChIP-seq data, as gene selection and clustering into YMC phases was done on the transcriptomics data.

Regarding histone modifications, N-PLS loading plot (Figure 4C) revealed that the two genomic regions defined to quantify each histone modification (–300 bp to TSS and TSS to +300 bp) were highly correlated, which suggests that a unique region is informative of the chromatin state. In addition, the histone marks plot also indicated that no batch effects or outliers were noticeable in the data (Supplementary Figure 3). Interestingly, N-PLS results also pointed out the relevance of H3K9ac and H3K18ac histone modifications on global gene expression regulation, since the highest loadings in absolute value were obtained for these marks. H3K4me3 was the least important histone modification in global terms, while H3K14ac, H3K56ac and H4K5ac presented intermediate relevance (phases in which these histone modifications peak are displayed in Supplementary Figure 4).

### 3.4. MORE Models Confirmed the Relevance of H3K9ac and H3K18ac

**Figure 5:**
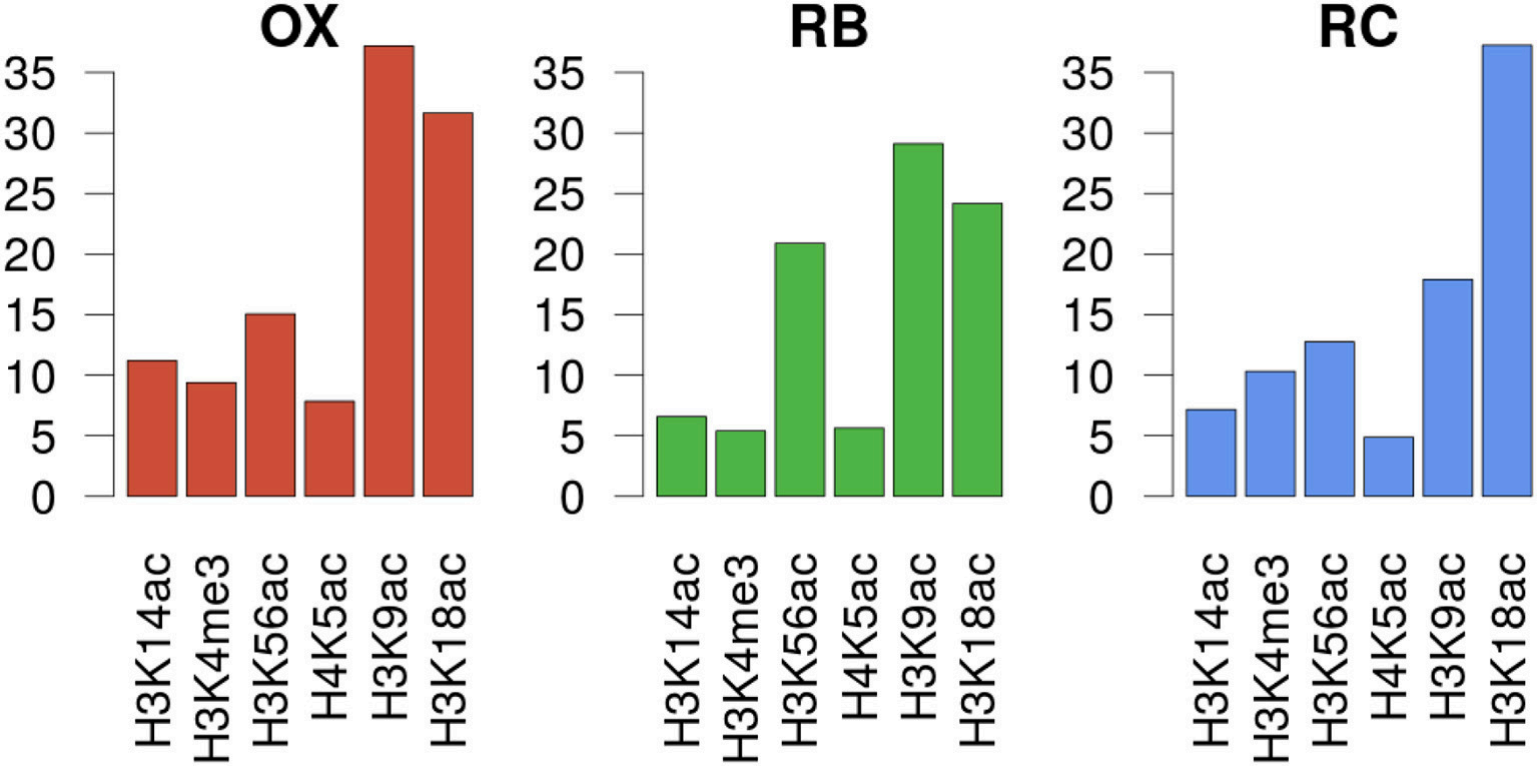
Gene expression regulation by histone modifications per YMC phase. Y axes show the percentage of genes in the cluster that was significantly regulated by each histone modifiation according to MORE models.

In order to unravel the specificity of the histone mark-gene expression regulation, MORE regression models were calculated for each gene using as predictor variables the normalized read count values at the genomic regions defined for each histone mark ChIP-seq assay. We decided to use only significant regulations having a positive regression coefficient, based on previous studies showing that histone modifications have a positive regulatory role on transcription activation (Berger, 2007). MORE results confirmed the relevance of H3K9ac and H3K18ac, as they appear as significant in the highest number of gene models, 780 and 815 respectively. H3K56ac, significantly associated to 393 genes, followed. When analysing the significant regulations per cluster (Figure 5 and Supplementary Figure 5), we found that H3K9ac regulates the highest proportion of genes in OX and RB phases (37 and 29%, respectively), followed by H3K18ac (31 and 24%). Notably, H3K56ac regulates 21% of genes in the RB cluster, a higher proportion than in the other clusters (13–15%). In the RC cluster, H3K18ac is the most significant histone modification, regulating 37% of the genes.

**Figure 6:**
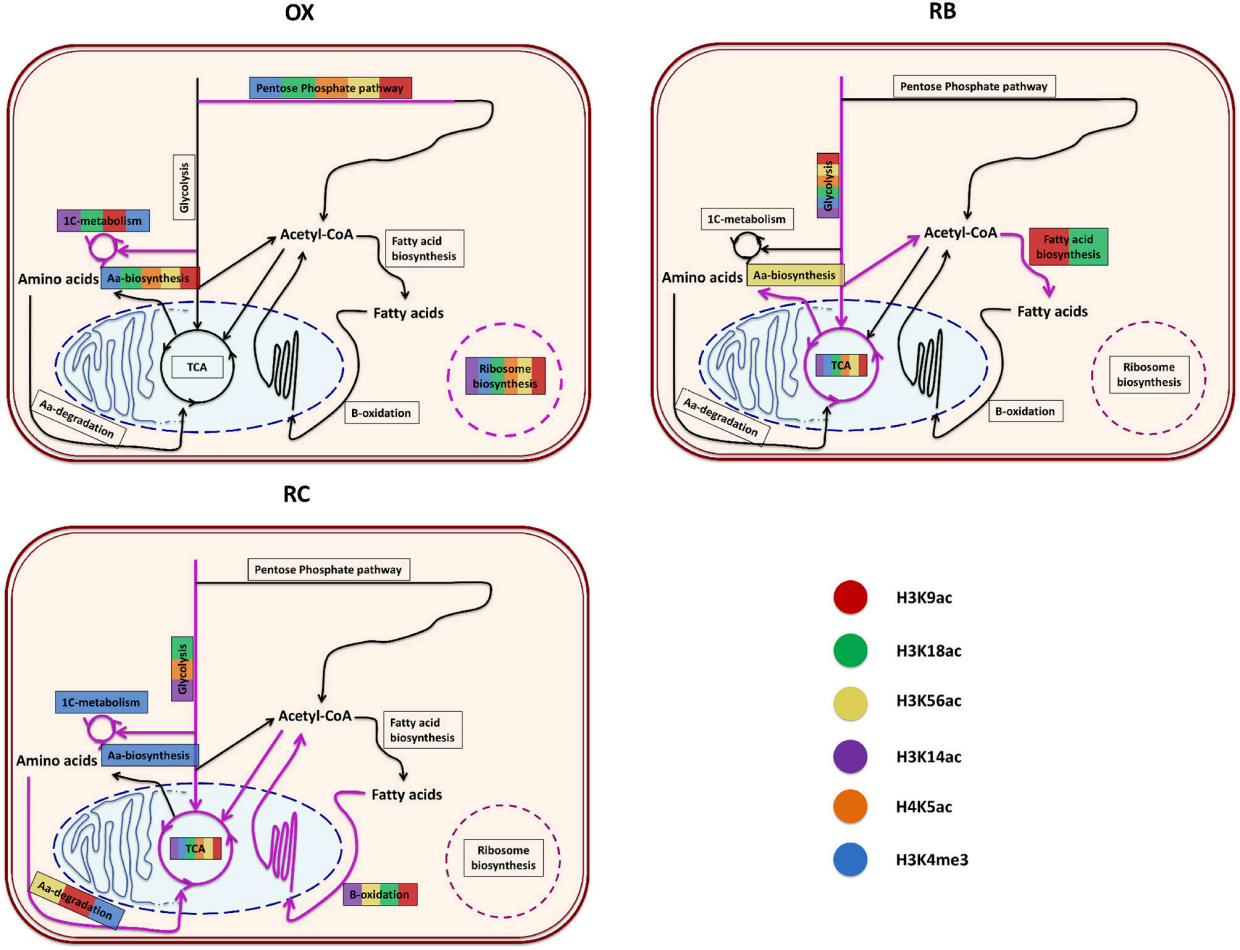
Functional enrichment of the genes that were significantly regulated by each histone modification according to MORE results. For each YMC phase, a diagram summarizes the enriched processes found for each color-coded histone modification. The order or the length of the colored lines present in a particular process has no special meaning, just that the processed was significantly enriched for those histone modifications. The diagrams represent the core metabolic pathways enriched for the different chromatin modifications.

In order to understand the impact of histone modifications on the regulation of cellular functions associated to each YMC phase, a functional enrichment analysis of the genes significantly regulated by each histone mark according to MORE was performed separately for each cluster. The results per phase are described next and illustrated in Figure 6 and Supplementary Figure 5.

#### 3.4.1. OX Phase

OX phase showed a common trend of regulating translation, ribosomal machinery and nucleotides metabolism. Except H4K5ac, every other histone modification was involved in amino-acid metabolism. H3K9ac, H3K18ac, H3K14ac and H3K4me3 were specifically linked with one-carbon metabolism and methylation through the terms *Methyltransferase activity, Asparagine biosynthetic process or Glutathione metabolism*. H3K56ac, H3K18ac and H4K5ac showed overrepresentation of *helicase activity* genes. H3K18ac and H4K5ac were also enriched in terms related to cell cycle regulation (*Cell division* and *Mitotic recombination*) but, contrary to our expectations, this was not the case for mark H3K56ac, previously reported to be linked to histone deposition in S-phase (Li et al., 2008).

#### 3.4.2. RB Phase

RB phase was characterized by the regulation of sugar metabolism, which is associated to all histone modifications, coupled with a regulation of mitochondrial activity, from which H3K4me3 and H3K56ac were excluded. Genes involved in phospholipid metabolism were enriched in H3K9ac and H3K18ac regulations, while cell cycle appeared to be controlled by H3K18ac and H3K4me3 marks. H3K9ac, H3K14ac and H4K5ac were significantly enriched in *Biosynthesis of secondary metabolites* functionalities.

#### 3.4.3. RC Phase

This phase was arguably the most diversified among the histone modification regulatory landscape. The tricarboxylic acid (TCA) cycle was regulated by all modifications through *Oxidative phosphorylation* and *Electron transport chain*, whereas Glycolysis was only enriched at H4K5ac, H3K18ac and H3K14ac. H3K14ac and H3K4me3 appeared to coordinate the regulation of ethanol metabolism genes, while H3K9ac associated to fatty acid metabolism together with H3K18ac, H3K56ac and H3K14ac. H3K18ac and H3K56ac combined to target genes within *cell division* functionalities, while H3K56ac, H3K9ac and H3K4me3 were related to amino-acid degradation processes. H3K56ac seemed to be the only mark associated to genes involved in *Histone acetylation* while H3K4me3 the only histone modification enriched in one-carbon metabolism genes.

## 3.5. MORE Identifies Most Relevant TFs for YMC Regulation

TFs are believed to work tightly with histone modifications to regulate gene expression. We used the MORE approach to identify key TFs of the YMC that complement histone mark regulation of gene expression. According to Yeastract database (Teixeira et al., 2017), 109 transcription factors were present among our DEGs. Yeastract database also provided the potential target genes for each TF, and this information was used to calculate MORE regression models. MORE results indicated that 105 TFs were significantly associated to 2480 genes. TFs that were regulating a significantly higher proportion of genes in the MORE models compared to Yeastract annotations were selected for further analysis. These TFs included Sfp1, Hfi1, Asg1, Ppr1, Ste12, Ylr278c, Cup9, and Dat1 at OX-phase, Yhp1 at the RB-phase, and Mig2, Pip2, Xbp1, and Cin5 at the RC-phase, making a total of 13 significantly over-represented (FDR adjusted *p*-value < 0.05) TFs within MORE models.

**Figure 7:**
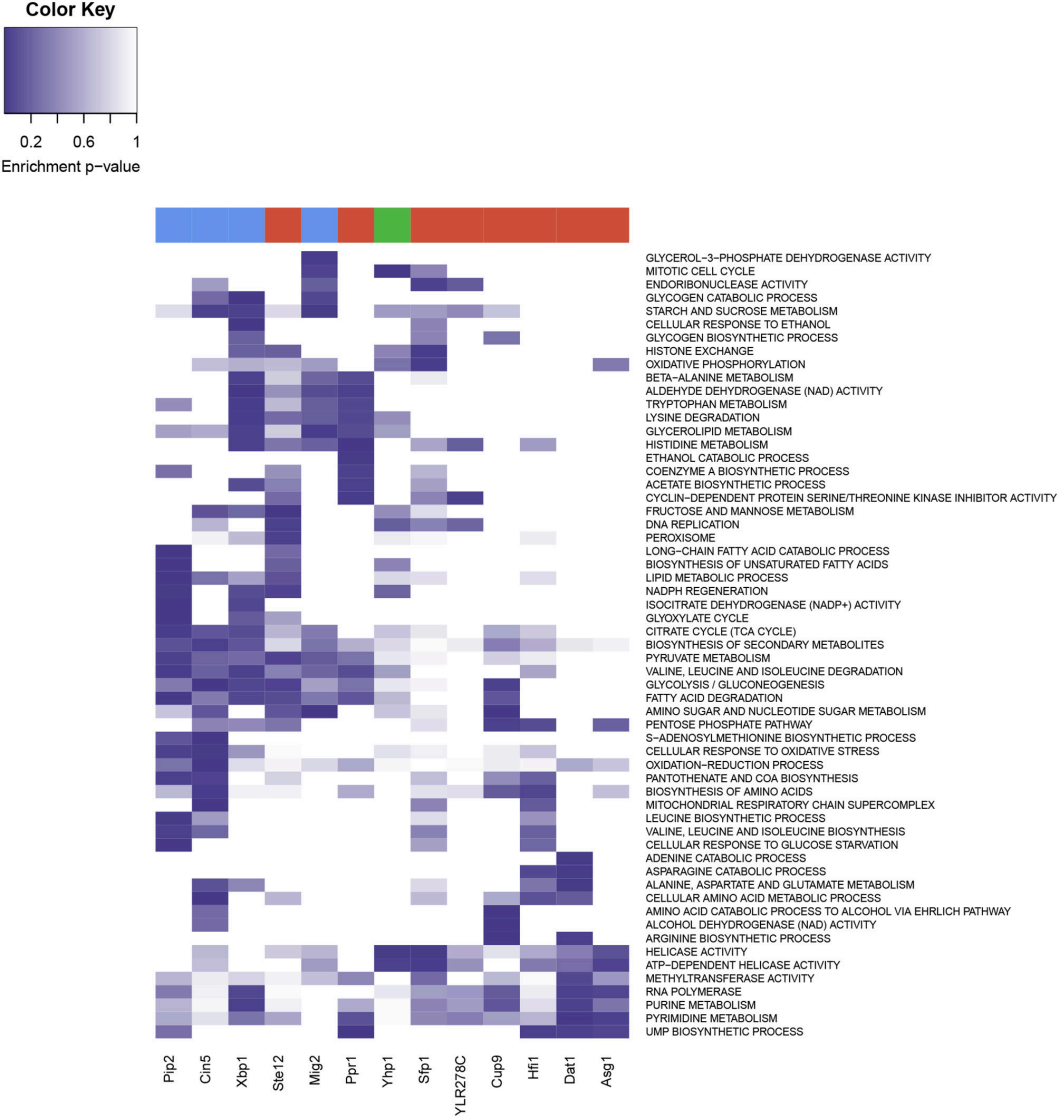
Heatmap from the functional enrichments of Transcription Factors. Rows display the different enriched GO and KEGG terms, and color intensity represent the *p*-value of the significance test. Transcription factors are present in the columns, at the top of each column three colors represent the three YMC phases (red for OX, green for RB and blue for RC).

A functional enrichment analysis on the genes significantly regulated by each of these 13 over-represented TFs (Figure 7) showed that these genes were involved in functions related to metabolic sensing, chromatin structure, and *cell cycle regulation*. Sfp1 appeared to be involved in the transcription of *ribosome*, nutrient response, *G2/M transitions*, DNA damage, and histone exchange. Hfi1 played a role in translation, aminoacid and nucleotides biosynthesis and nucleotide cleavage via *Helicase activity*. Pip2 was associated to lipid metabolism, amino-acid and carbohydrate metabolism and *citrate cycle*. Mig2 contributed to carbohydrate and amino-acid metabolism as well as *TCA cycle*, and was involved in endonuclease cleavage, *mitotic cell cycle* and *endocytosis*. Yhp1 enriched functions were *ribosome, helicase activity* and cell cycle regulatory control. Xpb1 and Ppr1 were involved in the cell cycle via cyclin regulation, but also in *ethanol metabolism* and showed regulation of various metabolic pathways, including carbohydrate and amino-acids metabolism. Cin5 was involved in stress response and metabolic response, in the context of the YMC this is mostly related to redox changes, and the regulation of glycolysis, and amino-acid metabolism. Ste12 was also involved in the *DNA replication* and fatty acid metabolism, Cup9 showed enrichment of glycolysis-related functionalities and Dat1 was involved in metabolic regulations in response to hipoxia, with nucleotide metabolism upregulated, which was also linked to Asg1. Ylr278c showed association with chromatin remodeling functionalities (*RSC complex* among others), as well as *Helicase activity* and other nucleotide repairing properties.

## 3.6. Gene Expression Co-Regulation by Histone Modifications and TFs

After separately studying the role of histone modifications and TFs on gene expression regulation, the results were combined to search for co-regulatory activity between these two types of regulation. First, an independence test (section 2.8) was applied to study if a given pair TF-histone modification was significantly present in a common set of genes. TFs considered for this analysis were the 13 over-represented factors previously described. Three of all the tested combinations gave significant results: histone modification H3K18ac associated with transcription factors Hfi1, Pip2 and Xbp1 (FDR adjusted *p*-values < 0.05).

**Figure 8:**
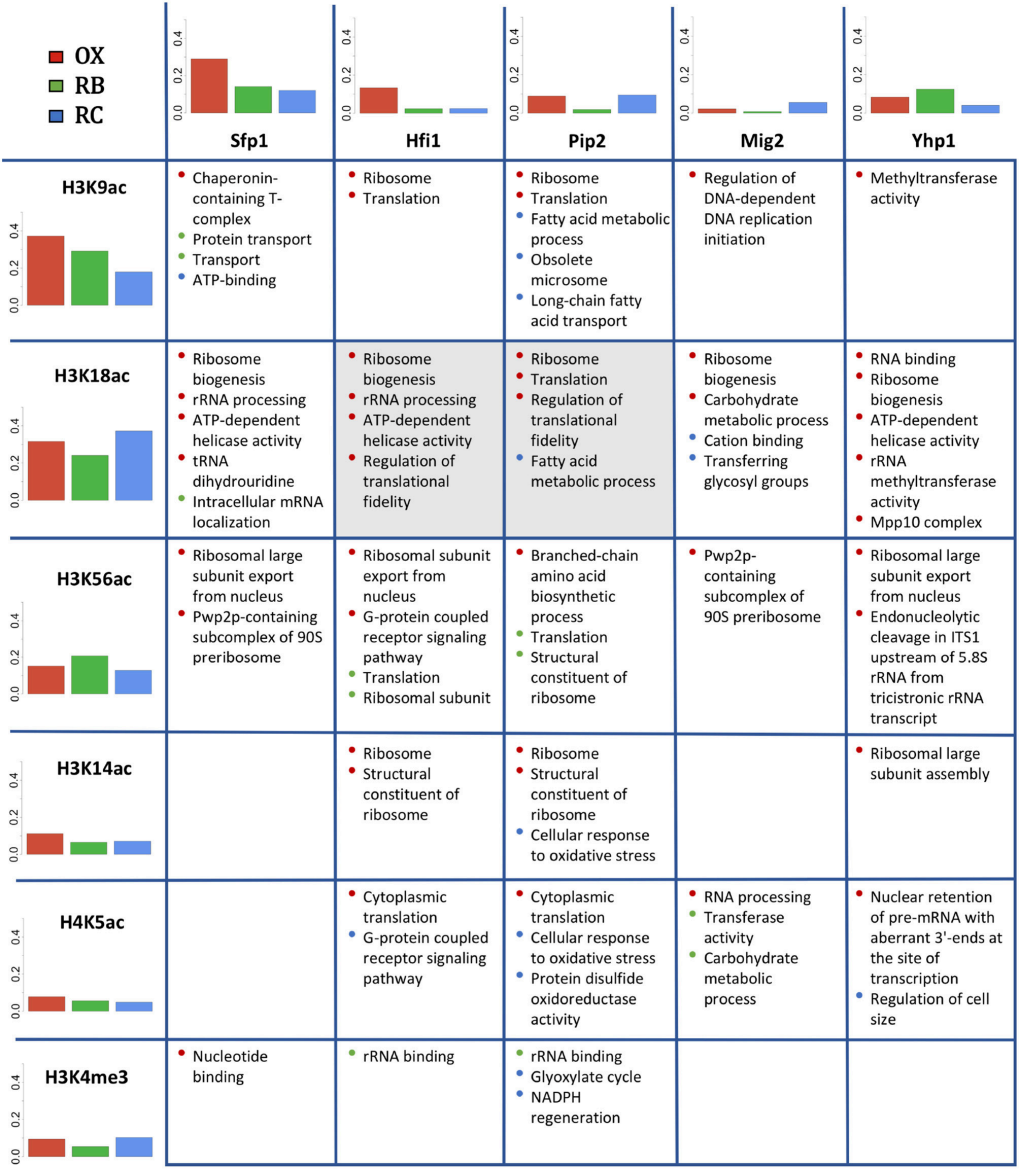
Co-regulation study for histone modifications and TFs. In rows, the studied histone modifications. In columns, the 5 most relevant TFs. Bar plots show the proportion of genes significantly regulated by each histone modification or TF within each cluster (YMC phase). The table displays the common enriched functional terms for each pair TF-histone modification. The color of the bullets refers to the cluster for which the functional term was enriched. Cells in gray color indicate that the corresponding pair TF-histone modification was co-regulating a significant number of genes (see section 3.6).

Next we compared the functional enrichment results obtained independently for histone modifications and TFs (Figure 8). For this analysis, we compared the 5 most relevant TFs (FDR adjusted *p*-value < 1.1*e* − 9) with all studied histone modifications. Figure 8 includes bar plots indicating the proportion of genes regulated within each YMC phase by each regulator in the table. In general, it is expected a higher proportion of regulated genes for histone modifications. This is simply due to the fact that all genes have their corresponding histone modification measurement but not all the genes have an associated TF according to Yeastract database. In OX phase, we found a relative higher number of genes regulated by Sfp1 and Hfi1 together with H3K9ac and H3K14ac. For RB phase, Yhp1 and H3K56ac seemed to be more important, while the number of significantly regulated genes for Mig2 and H3K18ac was higher in RC phase.

Comparative functional analysis revealed functionalities shared between histone modifications and TFs, most of which were consistent with the functions individually attributed to each regulatory element. For instance, H3K9ac, H3K18ac and H3K56ac share OX phase-related functionalities with all the TFs, including *Ribosome biogenesis, Translation* or *Helicase activity* among others. H3K14ac also shared some of these functionalities but only with TFs Hfi1, Pip2 and Yhp1. RB-phase did not show high overlap between histone modifications and TFs associated processes, H3K56ac shared *Ribosomal* and *Translation* functions with Hfi1 and Pip2, while H4K5ac shared *Carbohydrate metabolic process* with Mig2. Pip2 seemed to be the TF with the highest functional cooperation with histone modifications in RC-phase, showing common functionalities with H3K9ac, H3K18ac, H3K14ac, H4K5ac and H3K4me3, that include *Fatty acid metabolic process, Long-chain fatty acid transport* and *NADPH regeneration* among others.

## 4. Discussion

Temporal profiles of gene expression along the YMC have been widely studied by different authors (Klevecz et al., 2004; Tu et al., 2005; Slavov et al., 2011). However, little is still known about the regulatory effect of histone modifications on gene expression in the YMC. The pioneering study of Kuang et al. (2014) analyzed chromatin state data and its impact on gene expression in YMC. Their correlation analysis showed an association between histone modifications and YMC phases, but did not identify which particular genes were significantly regulated by each histone mark.

In this work, we recovered RNA-Seq and ChIP-Seq data from Kuang et al. (2014) study, and complemented them with an additional histone modification (H3K18ac). We re-processed ChIP-seq samples to obtain comparable chromatin state measurements, and redid the RNA-seq differential expression analysis to refine the clustering of the genes into the three YMC phases. We applied, for the first time in this context, an integrative strategy based on multivariate regression models that allowed us to elucidate interplay of histone modifications and TFs on the modulation of gene expression during the YMC.

The differential gene expression analysis was the starting point of the study and 2552 differentially expressed genes obtained with maSigPro method constituted the set of genes used for downstream analyses. The clustering of these genes into the three YMC phases (Figure 2) outperformed the previous clustering efforts (Kuang et al., 2014) according to the Silhouette quality indicator (Supplementary Figure 2), hence providing a solid landscape to conduct the omics integration analyses.

The multi-omic exploratory approach using a N-PLS multi-way regression model (Figure 4) confirmed the data was free of outliers and batch effects, and also the expected distribution of time points and genes in concordance with the YMC phases. An important conclusion that can also be drawn from the N-PLS results for histone modifications is that H3K9ac and H3K18ac were the main marks involved in changes of gene expression. Interestingly, these changes were mostly explained by the components that best separated the RC phase from the other YMC phases. As these differences corresponded to the separation of high and low oxygen consumption, it could be hypothesized that chromatin changes have the highest impact on gene expression through the change in cellular redox state. These results are in line with a YMC division into two phases (HOC and LOC) based on the oxidative state of the cell as proposed in Mellor (2016).

While N-PLS allowed to globally explore the relationship between gene expression and histone modifications, the MORE method provided the framework for studying the specifics in the regulation of the expression of each particular gene. Hence, MORE generalized linear models identified which histone marks and TFs significantly associated to each gene along the YMC. MORE models confirmed the N-PLS results that revealed H3K18ac and H3K9ac as histone modifications with the highest impact on gene expression, since they were significantly regulating a larger proportion of genes with cycling transcripts. While both of them show a similar relevance in OX and RB phases, H3K18ac alone gains importance in the RC phase. We can also highlight here the contribution of H3K56ac to the regulation of the RB cluster. Additionally, our analyses highlighted a set of 13 TFs as key for the YMC regulation. We hypothesize that combination of histone modifications and TFs are capable of shaping the YMC, and propose they act cooperatively across the cycle to contribute to gene expression regulation needs.

The functional enrichment analysis revealed that H3K9ac and H3K18ac target genes were mostly involved in ribosome activity and translation in OX phase, in mitochondrial activity and glycolysis in RB phase, and in fatty acid degradation during the RC phase. Results for H3K9ac are in agreement with those reported in Kuang et al. (2014) (H3K18ac was not included in that study), which were derived from the functional enrichment of genes within each YMC phase. Thus, our findings support these two histone marks as primarily responsible for driving clusters functionalities. Previous studies have pointed to the relevance of acetyl-CoA in coordinating gene expression through protein acetylation levels (Wellen et al., 2009), which peak toward the end of OX phase (Cai et al., 2011). This correlates with positive regulation of CHO metabolism as cells enter the RB phase. Interestingly, although activation of glycolysis has been linked to acetylation of genes involved in this metabolic pathway, the RB phase presents the lowest number of acetylated genes in the YMC.

Association analysis between histone modifications and TFs suggested Pip2 and Hfi1 to combine with H3K9ac and H3K18ac to drive the OX phase. Interestingly, H3K56ac also shared ribosome functionalities with Sfp1, Hfi1, Mig2 and Yhp1 in the OX phase, and translation functionalities with Hfi1 and Pip2 in RB phase. This putative function in transcription control is likely to be related to its role in histone turnover at promoters (Rufiange et al., 2007) whereas H3K56ac role in cell division in the RC phase is more associated to its role in compaction of DNA into chromatin following DNA replication and repair (Kurdistani and Grunstein, 2003), linked to peak levels of H3K56ac in S phase of the cell cycle; the role of H3K56ac in replication was previously linked to the RB phase of the YMC (Cai et al., 2011). Pip2 is the main regulator of RC phase in coordination with the histone modifications (Figure 8), which presumably drive the fatty acid metabolic capabilities of this phase, consistent with the fatty acid response functionalities associated to Pip2 (Karpichev et al., 1997; Baumgartner et al., 1999). Overlapping functionalities for H3K9ac and H3K18ac with Pip2 revealed a possible coordination of the marks and the transcription factor to drive fatty acid metabolic processes in RC phase, while Pip2 association with H3K14ac, H4K5ac and H3K4me3 is more consistent with the response to changes in redox conditions.

Co-regulation analysis revealed that H3K18ac shared a significant number of genes precisely with Pip2 and Hfi1. Pip2 triggers the regulation of genes required for beta-oxidation (Karpichev et al., 1997), while Hfi1 is required for the acetylation mechanisms of SAGA complex. Thus, our results capture the central concept of the YMC, the link between metabolism and the mechanism for driving chromatin structural changes via lysine acetyl transferases leading to the expression of genes that drive metabolism. This can also be observed at the H4K5ac and Mig2 pair, which share the common function of *carbohydrate metabolic process* (Figure 8), which is an important RB phase pathway, possibly driven by the glucose-response activities previously described for Mig2 (Fernández-Cid et al., 2012). Pip2, Xbp1, Cin5, Ste12, and Cup9 also showed involvement in metabolic response, mostly through fatty acid and glucose metabolism, but they were not associated with any of the histone modifications. Other major cell functions such as the cell cycle were associated with transcription factors (Yhp1, Asg1, Ste12, Cin5) and histone modifications (H3K18ac, H3K56ac, H4K5ac), although no associations between them were detected.

Although H3K4me3 was not associated with the regulation of a high number of genes (Howe et al., 2017), it is worth mentioning this modification is associated to genes involved in synthesis of amino acids, and specifically one-carbon metabolism. Histone methylation dynamics have been related to availability of methionine and regulation of one-carbon metabolism, which in turn is the main supplier of S-adenosyl methionine for protein methylation (Mentch et al., 2015). H3K4me3 did not show a substantial overlap with any of the TFs, which might suggest a role independent from the selected TFs.

All together, the present study reveals the interplay between transcription factors and histone modifications in the regulation of YMC gene expression. The usage of multivariate and regression models has helped to improve the conclusions gathered in previous studies with more detailed functional characterization of the YMC, and allowed to identify H3K9ac and H3K18ac as the main histone modification drivers that define this biological cycle, being the main contributors to the regulation of fatty acid and aminoacids metabolism, glycolysis and TCA. The present study also identified 10 TFs that may be important for YMC regulation. Many associations were found between cellular functions enriched at TFs and histone mark regulatory models, offering a possible landscape in which these two molecular levels might cooperate to drive changes in gene expression that shape the YMC phases. Although metabolomic data was not analyzed here, we were able to capture the relationship between histone modifications and cellular metabolic state through functional analyses, suggesting that a future incorporation of metabolomics and chromatin accessibility data will contribute to further understand the set of molecular regulatory mechanisms behind the YMC.

## Data Availability Statement

H3K9ac, H3K14ac, H3K56ac, H3K4me3, and H4K5ac ChIP-Seq datasets and gene expression data can be accessed from GEO repository GSE52339. H3K18ac ChIP-Seq data can be accessed at GEO repository GSE118889.

## Author Contributions

VS-G processed histone modifications datasets, modeled gene expression data, applied N-PLS and MORE regression methods, contributed to co-regulation and functional enrichment analysis, created some of the figures and participated in manuscript drafting. SC-G contributed to data processing, integrative analysis, functional and co-regulation analysis, biological interpretation, creation of the figures and wrote the manuscript. MU contributed to data integration analyses. ZK conducted the data collection. JM studied concept, collaborated in the biological interpretation of results, and reviewed the manuscript. AC studied concept, designed the analysis strategy, supervised work, and drafted the manuscript. ST designed the integrative analysis strategy, supervised work, created some figures and wrote the manuscript.

### Conflict of Interest Statement

SC-G is employed by company BioBam Bioinformatics S.L., AC is co-founder in the company. The remaining authors declare that the research was conducted in the absence of any commercial or financial relationships that could be construed as a potential conflict of interest.
