# Supplementary material for: Elucidating the Role of Chromatin State and Transcription Factors on the Regulation of the Yeast Metabolic Cycle: A Multi-Omic Integrative Approach
Source: Front Genet. 2018 Nov 30;9:578. doi: 10.3389/fgene.2018.00578 (PMC6284056; doi:10.3389/fgene.2018.00578)
Supplement: Supplementary file 1 [file Data_Sheet_1.PDF]

Elucidating the regulation of the Yeast Metabolic Cycle through the integration of gene expression and chromatin status

Supplementary material

Figure 1. PCA on differentially expressed genes. Score plot from the PCA performed with the 2552 differentially expressed genes.......... 3
Figure 2 Silhouette plot of the gene clustering in this project (up) and in (Kuang, et al., 2014), (bottom). RC genes (blue), RB (green) and OX (red). 4
Figure 3. Histone modification loading plot colored and shaped according to batches and sequencing technologies. 5
Figure 4. Histone modification loading plot colored according to the phase of the higher proportion of associated genes..........6
Figure 5. Heatmap from functional enrichments of the histone modifications target genes.......7

Table 1. Elements of the core sorted by the percentage of explained variation. 5

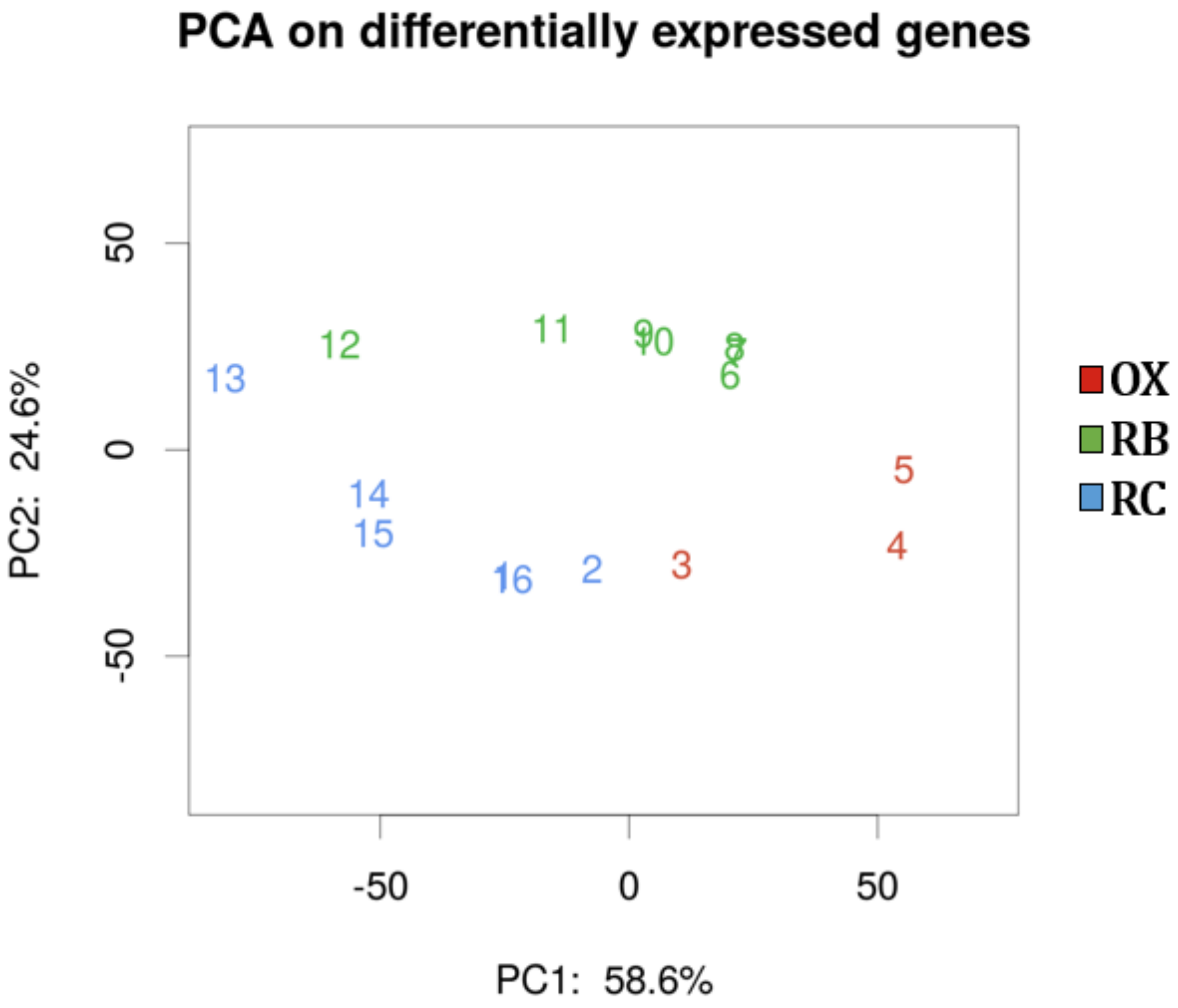

Figure 1. PCA on differentially expressed genes. Score plot from the PCA performed with the 2552 differentially expressed genes.

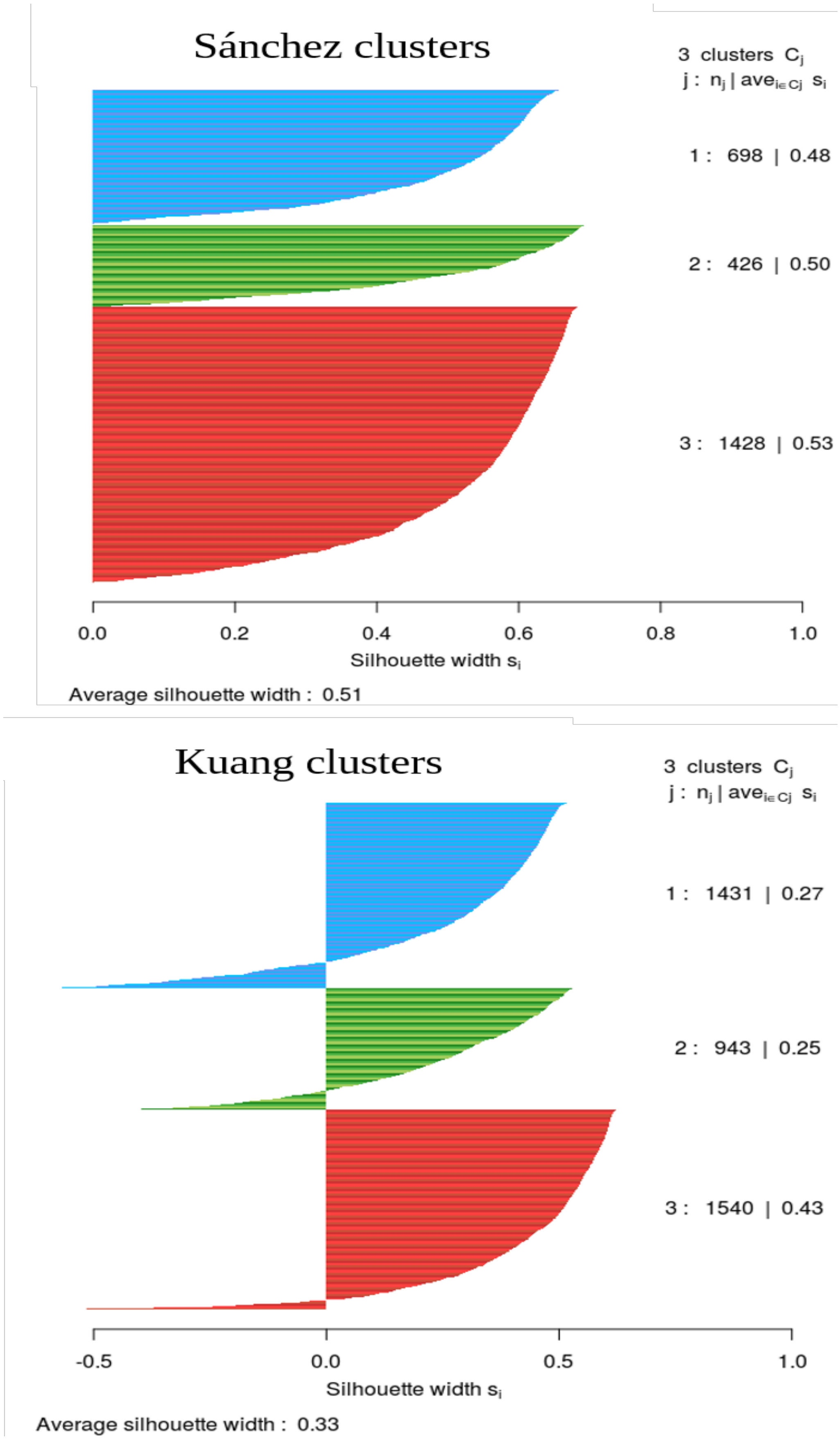

Figure 2 Silhouette plot of the gene clusters from this project (up) and from (Kuang, et al., 2014), (bottom). RC genes (blue), RB (green) and OX (red).

Table 1. Elements of the core sorted by the percentage of explained variation.

	Core element	Explained variation of the core
1	(2,2,2)	60.69%
2	(2,2,1)	23.92%
3	(1,1,2)	5.04%
4	(1,1,1)	4.64%
5	(1,2,1)	2.69%
6	(1,2,2)	2.47%
7	(2,1,2)	0. 34%
8	(2,1,1)	0.21%

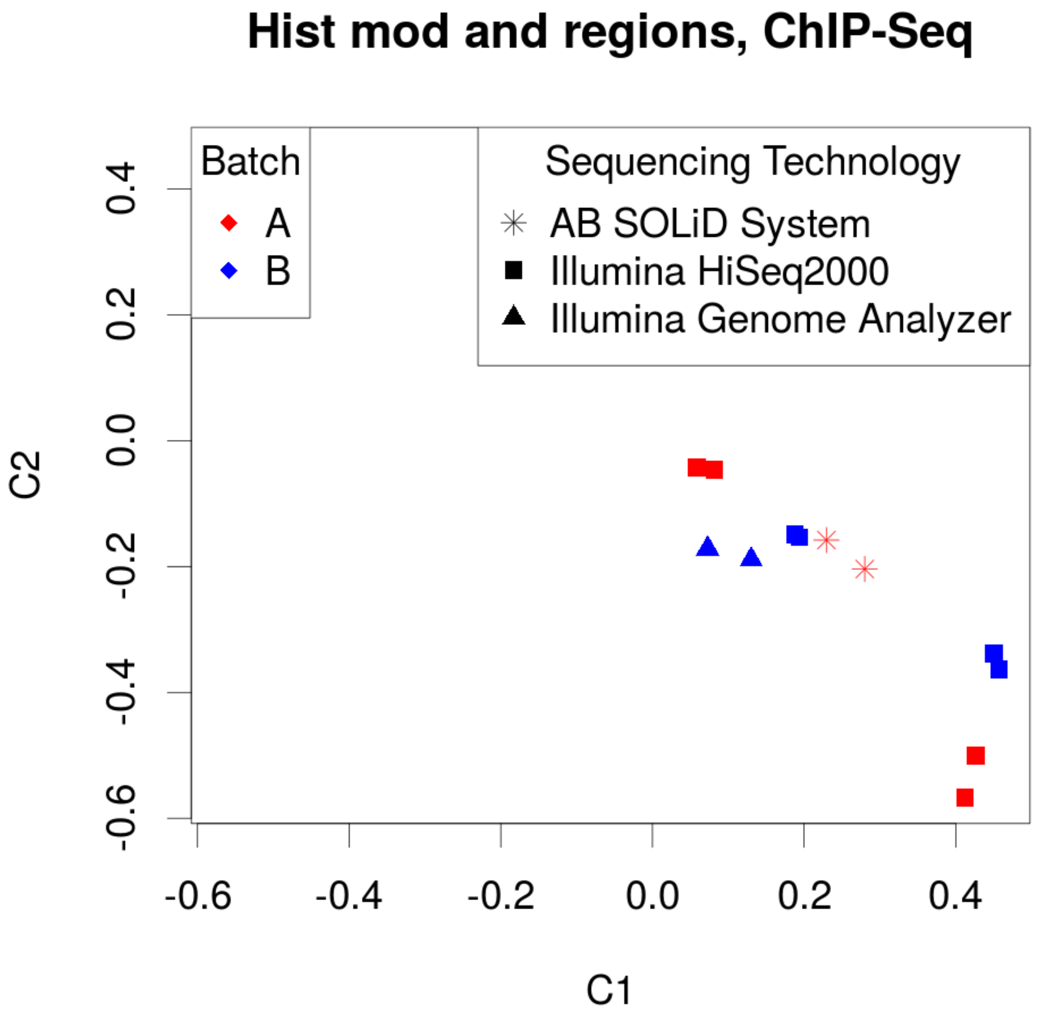

Figure 3. Histone modification loading plot colored and shaped according to batches and sequencing technologies.

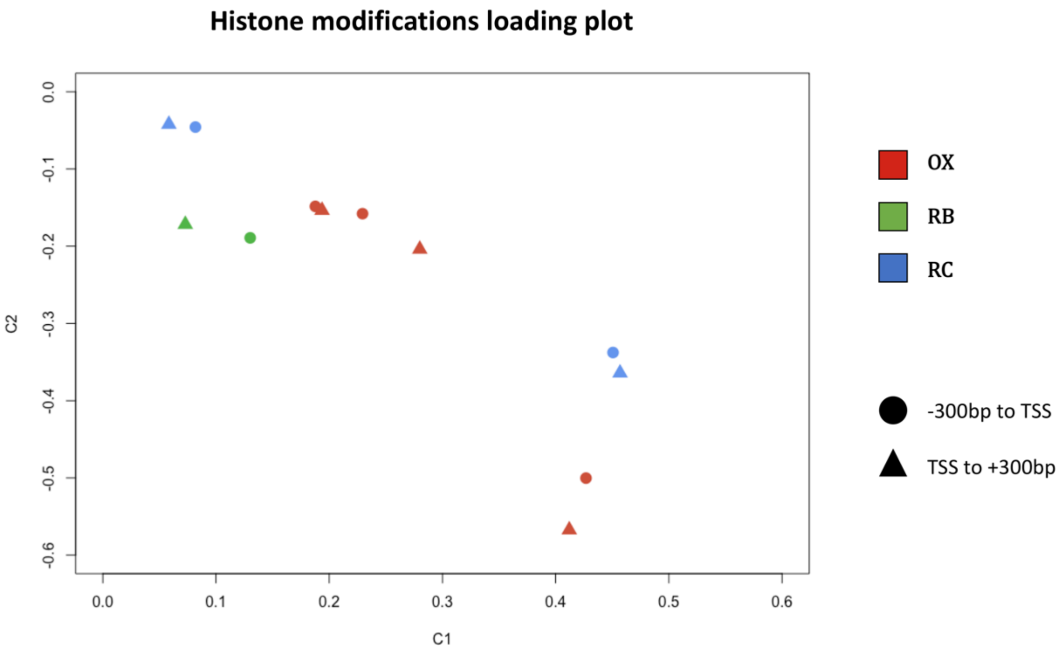

Figure 4. Histone modification loading plot colored based on the phase of the cycle on which the given histone modification has a higher proportion of associated genes.

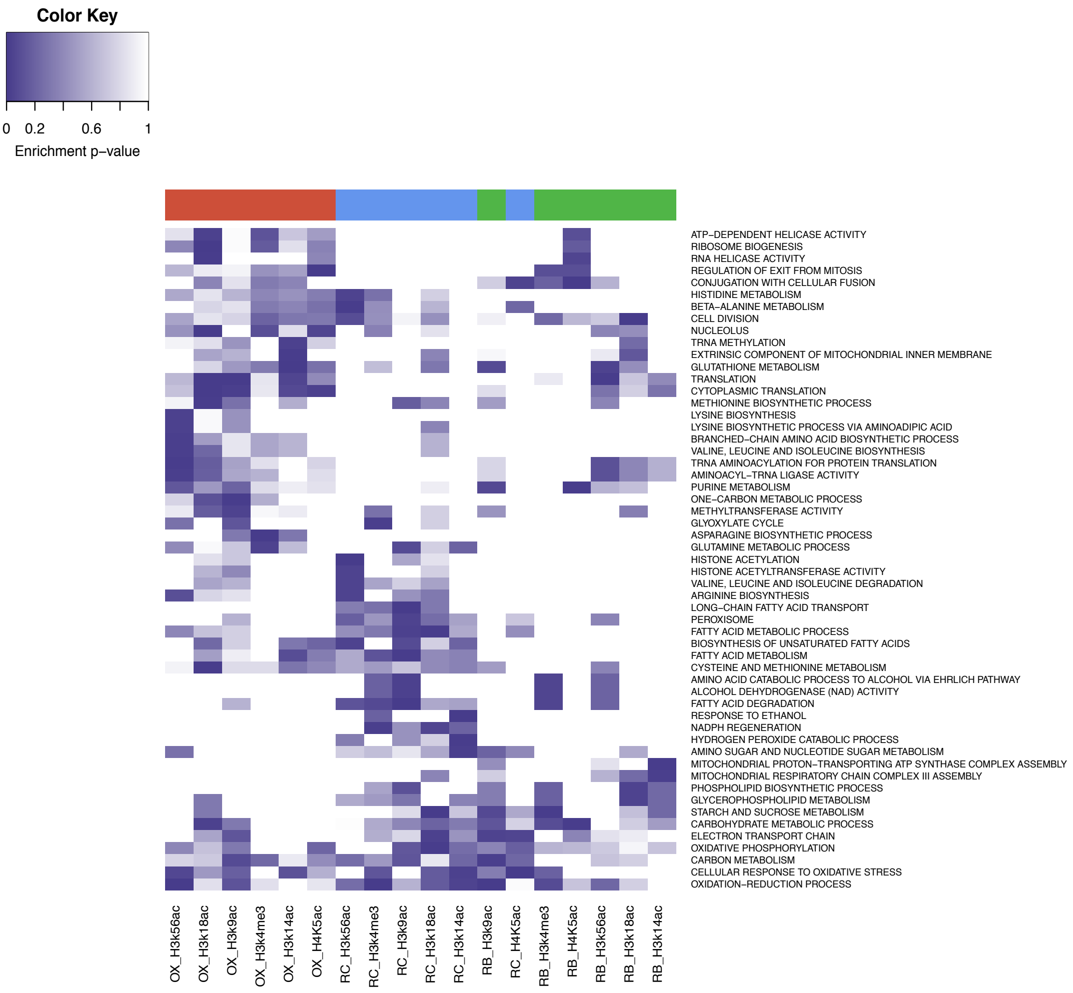

Figure 5. Heatmap from the functional enrichments of Histone modification target genes from each YMC phase. Rows display the different enriched GO and KEGG terms, and color intensity represent the p-value of the significancy test. Histone modifications per phase are present in the columns, at the top of each column three colors represent the three YMC phases (red for OX, green for RB and blue for RC)
